# Wall Shear Stress Measurements Based on Ultrasound Vector Flow Imaging: Theoretical Studies and Clinical Examples

**DOI:** 10.1002/jum.15253

**Published:** 2020-03-03

**Authors:** Yigang Du, Alfredo Goddi, Chandra Bortolotto, Yingying Shen, Alex Dell'Era, Fabrizio Calliada, Lei Zhu

**Affiliations:** ^1^ Shenzhen Mindray Bio‐Medical Electronics Co., Ltd. Shenzhen China; ^2^ SME Medical Center Diagnostic Imaging Varese Italy; ^3^ Radiology Department Fondazione Istituto di Ricovero e Cura a Carattere Scientifico, Policlinico San Matteo Pavia Italy

**Keywords:** Doppler ultrasound, nonlaminar flow, turbulence, vector flow imaging, wall shear stress

## Abstract

Wall shear stress (WSS) is considered as a key factor for atherosclerosis development. Previous WSS research based on pulsed wave Doppler (PWD) showed limitations in complex flows. To improve accuracy for nonlaminar flow, a commercial ultrasound vector flow imaging (UVFI)‐based WSS calculation is proposed. Errors for PWD are presented theoretically when flow is not laminar. Based on this, simulations of WSS calculations between PWD and UVFI were set up for different turbulent flows. Our simulations show that UVFI has obviously better performance than PWD in WSS calculations. Wall shear stress results in different flow conditions at carotid bifurcations are described.

**Abbreviations**
2D: 2‐dimensional
CB: carotid bifurcation
PWD: pulsed wave Doppler
US: ultrasound
UVFI: ultrasound vector flow imaging
WSR: wall shear rate
WSS: wall shear stress

Arterial diseases are the leading cause of death worldwide and can also be accompanied by severe long‐term disability.^1^, ^2^, ^3^ Despite the systemic nature and the multiple risk factors involved, the observation that atherosclerosis occurs at bifurcations or arterial branches has led to the hypothesis that blood flow characteristics, such as secondary flows and randomly and rapidly fluctuating velocities, are correlated with plaque development.^4^ Numerous studies attempted to discover the connection between hemodynamic features and cardiovascular disease.^5^, ^6^, ^7^, ^8^ According to current knowledge, the vessel wall is considered capable of detecting hemodynamic stimuli and releasing vasoactive substances to preserve the cross‐sectional luminal area.^9^ Local hemodynamic forces have been proposed to regulate the site‐specific development of atherosclerosis. One is the tensile stress exerted perpendicular to the vessel wall by the blood pressure. The other is the frictional force exerted parallel to the vessel wall by blood viscosity and is called “wall shear stress” (WSS). Flow and vessel diameter changes have a substantial influence on WSS values, which are less affected by the blood viscosity.^10^ The endothelium appears to have a central role. Although endothelial cells respond to tensile stress (ie, arterial pressure and vessel stiffness), WSS appears to be the primary determinant of endothelial cell function.^7^, ^11^ The vessel remodels itself in response to long‐term changes in flow and arterial pressure, such that the luminal diameter reshapes to maintain a constant predetermined level of WSS in response to the release of vasoactive substances, the levels of which are strongly influenced by shear stress.^12^, ^13^ However, in some conditions, this adaptive regulation is not sufficient, and vessel geometry may affect the WSS. Evidence shows that atherogenesis mainly involves the outer walls of vascular bifurcations and locations of flow recirculation and stasis.^14^ In the carotid bifurcation (CB), the flow mainstream moves upward along the flow divider. Also, depending on the vessel enlargement, rotating secondary flow moves upstream toward the outer wall of the internal carotid artery. At that level, the velocity vectors have directional changes (ie, oscillations), which result in unsteady WSS, and the arterial wall has mean flow shear stress that is considerably lower in magnitude during the cardiac cycle. These flow characteristics are absent from vascular regions spared from atherosclerosis.^14^, ^15^ Consequently, a steady or unsteady WSS within the vasculature plays a vital role in the long‐term health of the blood vessels.^15^, ^16^ A WSS assessment in clinical practice may be helpful in the analysis of diverse pathophysiologic conditions.

The investigation of blood flow, in bifurcation or vessel stenosis, requires an advanced flow‐measuring method, in which both the absolute magnitude of the velocity and flow direction should be obtained, for an optimal assessment of WSS. The quantitative measurement of WSS metrics for real clinical use is desired to be accurate and accessible under different conditions: eg, nonlaminar flow in large vessels and high‐velocity flow combined with low plasma viscosity, which may cause endothelial injury in small vessels.^17^

However, this goal has not been achieved for many years. Until now, the 2 imaging methods available to measure WSS were magnetic resonance imaging^7^, ^8^, ^18^, ^19^, ^20^ and maximum velocity–based or multigate pulsed wave Doppler (PWD) imaging,^21^, ^22^, ^23^, ^24^, ^25^ but neither of them has been able to provide a precise measurement. Magnetic resonance imaging is partially faulty, mainly because of resolution limits^26^ and nonlinear underestimation.^27^ Pulsed wave Doppler has limitations: first, because the measurements are based on the assumption that the flow is laminar; second, because of the limitation of angle dependence for conventional Doppler ultrasound (US)^28^; and last, because the flow estimation of low velocities near the pulsating arterial wall via PWD is challenging.^29^

This article introduces an ultrasound vector flow imaging (UVFI)‐based WSS measurement method. Compared to conventional US, the new method uses vector velocities derived from UVFI, a technique based on ultrafast imaging, which provides angle‐independent and multidimensional dynamic visualization of 2‐dimensional (2D) velocity vectors from multidirectional transmission and reception of plane waves^30^, ^31^, ^32^ with an interleaved emission sequence.^33^

Based on this technique, WSS around complex or turbulent flow can be measured with the proposed method for more advanced clinical studies of vascular disease. For example, WSS measurement might be helpful to identify patients at high risk of plaque development in the CB due to reversed or complex flow behavior or as a consequence of vessel geometry. The application of WSS measurement might result in advanced clinical management in the early stage of atheromasia. Moreover, abnormal WSS might be an indication of potential plaque breakage, thus allowing the detection of high‐risk plaques in noncritical stenosis.

The article is organized as follows: The theory is presented in the “Theory” section, where both the conventional and UVFI‐based WSS calculations are demonstrated. The results are simulated on the basis of the presented theory and compared to each other in the “Simulation Results and Discussion” section. Several typical clinical examples are shown in the “Clinical Examples and Discussion” section to demonstrate WSS‐measured findings under different flow types, which are obtained by using a commercial US system with the proposed UVFI‐based technique. Conclusions are drawn in the last section.

## Theory

The general equation for calculating WSS is defined by^34^, ^35^, ^36^

(1) $$\tau =\mu {\left.\frac{\partial v}{\partial r}\right|}_{\text{wall}},$$

where *μ* is blood viscosity, and the subscript “wall” denotes that the shear stress *τ* is measured on the vessel wall, where (*∂v*/*∂r*)_wall_ is also called the wall shear rate (WSR). Thus, WSS is a multiplication of blood viscosity and the WSR, in which *μ* varies with the WSR, since blood is non‐Newtonian fluid. However, the existing studies have stated that for significantly different WSRs, the variation of *μ* is trivial,^37^, ^38^ leading to a little difference in WSS measurement.^36^, ^39^ Therefore, a constant value for blood viscosity can be used: eg, *μ* = 3.5 mPa • s for carotid arteries.^24^, ^40^

**Figure 1:**
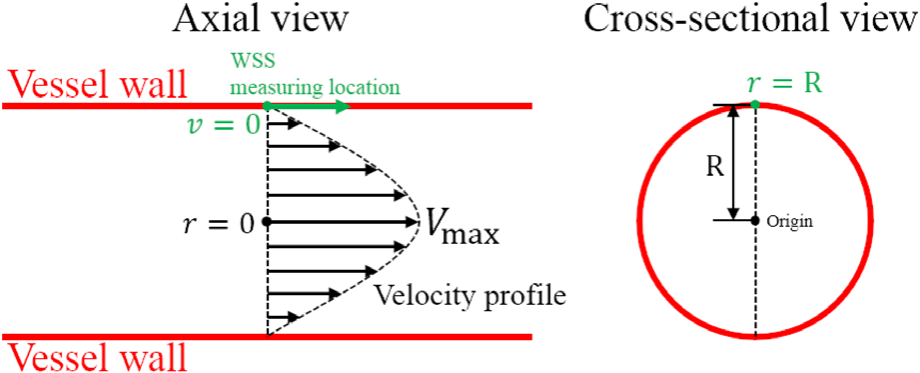
Wall shear stress measurement with an ideally shaped vessel and velocity profile. *R* is the radius of the vessel; *r* is the distance from the WSS measuring location to the origin, which is the center of the vascular cross‐section; and *V*_max_ is the maximum value of the velocity profile assumed to be parabolic.

The parameter *v* in Equation 1 is the velocity in parallel with the vessel wall, and *r* is the distance perpendicular to the wall, as illustrated in Figure 1, in which it is assumed that the vessel is cylindrical and straight and has laminar flow with a parabolic velocity profile given by

(2) $$v=\left|-kr^{2}+V_{\max}\right|.$$

Substituting Equation 2 into Equation 1 with *r* = *R* and *v* = 0 on the wall yields

(3) $$\tau =\frac{2\mu V_{\max}}{R}.$$

This is the conventional US way for measuring WSS and only valid for pure laminar flow, where *V*_max_ can be measured by the conventional PWD with angle correction. Note that for the WSS, *τ* has the same direction as the flow velocity *v*, as shown in Figure 1.

The WSS varies with time, as the flow velocities are not constant, so that Equation 3 can be rewritten as a function of time by

(4) $$\tau \left(t\right)=\frac{2\mu V_{\max}\left(t\right)}{R},$$

where *V*_max_(*t*) denotes the maximum velocities at different time instances, where PWD can be used for estimating *V*_max_ (*t*).

### *Advanced Methods With Conventional US*

Pure laminar flow does not exist in reality, and velocity profile skewing can widely be found in vessels even if they are considered long and straight.^24^, ^41^, ^42^, ^43^ This could make substantial errors up to 20% to 60% for the *V*_max_‐based WSS measurements depending on the degree of velocity profile skewing, as presented previously by Mynard et al.^24^

**Figure 2:**
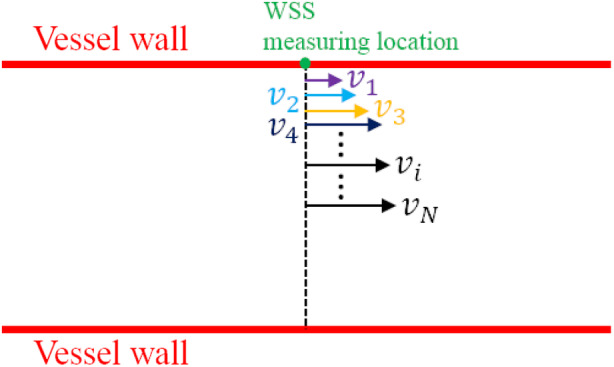
Wall shear stress measurement based on the multigate PWD, which is used to measure *v*_1_, *v*_2_, *v*_3_,…*v*_N_.

To avoid errors, multigate PWD is used, and WSS can directly be calculated with the general Equation 1, as shown in Figure 2, by which WSS as a function of time can further be formulated by

(5) $$\tau \left(t\right)=\mu \sum_{i=1}^{i=N}\frac{v_{i}\left(t\right)}{{\Delta r}_{i}},$$

where *Δr*_*i*_ is the distance from the *i*th velocity measurement of PWD to the WSS measurement location; *N* is the number of velocities used for WSS estimation; and *v*_*i*_(*t*) is the *i*th corresponding velocity at different time instances and can be obtained by multigate PWD with angle corrections. In an early study, Brands et al^23^ applied multigate PWD with a similar equation for calculating the WSR:

(6) $$\mathrm{WSR}\left(t\right)=\max_{1\leq i\leq N}\frac{v_{i}\left(t\right)}{{\Delta r}_{i}},$$

where the maximum value is considered the measured WSR. With Equation 5, 6, the WSS estimation will not be affected by the velocity profile skewing but is still angle dependent. Because many rouleaux do not travel exactly parallel to the vessel wall and may also change their directions at different times, the true velocities cannot be accurately estimated by angle correction, since the velocities from PWD originally denote only the velocity components along the direction of the US beam. On the other hand, the velocity used in the WSS estimation is the velocity component parallel to the vessel wall from the assumed true velocities that are directly obtained from PWD with angle correction. Therefore, in the end the incorrect velocities evaluations result in uncertain errors in the WSS estimation, which will be illustrated in the following section.

### *Errors From Doppler Angle Corrections*

**Figure 3:**
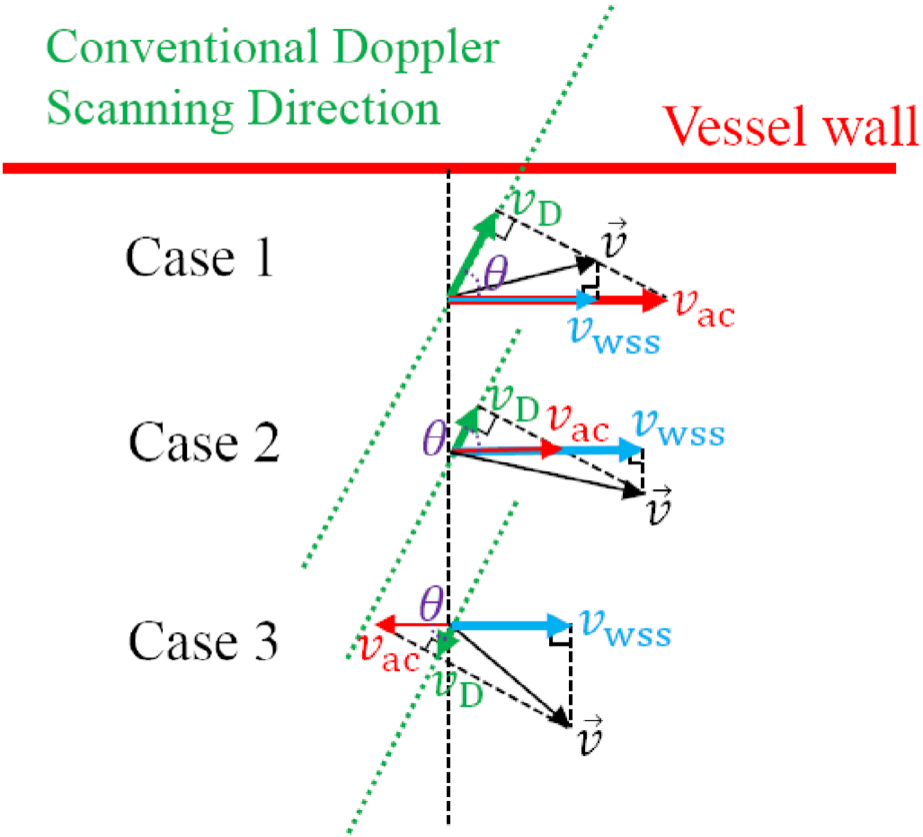
Presentation of differences between the angle‐corrected velocity *v*_ac_ and the velocity *v*_wss_ that should be used in the WSS estimation for 3 possible cases; *v*_ac_ is obtained by the conventional PWD with angle correction, where *v*_D_ is the result directly measured by PWD and *θ* is the beam‐to‐flow angle; *v*_wss_ is derived from the vector velocity $\vec{v}$, which is also considered the true velocity in the vessel.

The conventional US (PWD)‐based WSS measurement is restricted to the true velocity direction. It will give rise to errors if *v*_*i*_ in Figure 2 is not parallel to the vessel wall. This can be illustrated in Figure 3, where 3 possible cases are presented. The angle‐corrected velocity *v*_ac_ can be obtained by *v*_ac_ = *v*_D_/cos*θ*, where *v*_D_ is the velocity measured by conventional Doppler US (eg, PWD), and *θ* is the Doppler angle (the angle between *v*_D_ and *v*_ac_, also called beam‐to‐flow angle). The velocity used in the WSS estimation is *v*_wss_, which is parallel to the vessel wall and can be derived from the vector velocity $\vec{v},$ which is considered the true velocity possibly not parallel to the vessel wall even in a slightly curved vessel. For case 1 (Figure 3), the *v*_ac_ is bigger than the *v*_wss_, meaning that the *v*_wss_ will be overestimated by conventional PWD with angle correction. Likewise, the *v*_wss_ will be underestimated in case 2 and with an opposite wrong direction in case 3. The differences between *v*_ac_ and *v*_wss_ will result in uncertain errors in the WSS calculation using Equations 5 and 6.

Estimation of WSS only with PWD will completely fail for bending vessels with complex flows: eg, the CB, which is considered one of the most plaque‐growing places. To the best of our knowledge, there is still no clinical study of WSS for the CB only with conventional Doppler US. Tortoli et al^25^ proposed a dual‐beam method, which could find the real Doppler angle for velocities and thus estimate the WSR with multiple gates, but the major drawback is the inconvenience due to the simultaneous use of 2 transducers.

### *Ultrasound Vector Flow Imaging–Based WSS*

Ultrasound vector flow imaging is a novel technique^30^ in which both the magnitude and direction of the flow velocity can be obtained for the study of blood flow. Although the in‐plane or out‐of‐plane velocity component of the flow cannot be detected in the current 2D version, the vector (true) velocity on the 2D imaging plane can be accurately measured throughout the multiple‐angle approach used for US transmission and reception.^31^ The resulting velocity components along each angle are then combined by an angle‐compounding algorithm to calculate the vector velocity. The number of angles influences the accuracy of the estimated velocities.^44^ The UVFI technique has been implemented on a clinical US system^32^: Resona 7, which is a high‐end platform manufactured by Mindray (Shenzhen, China).

**Figure 4:**
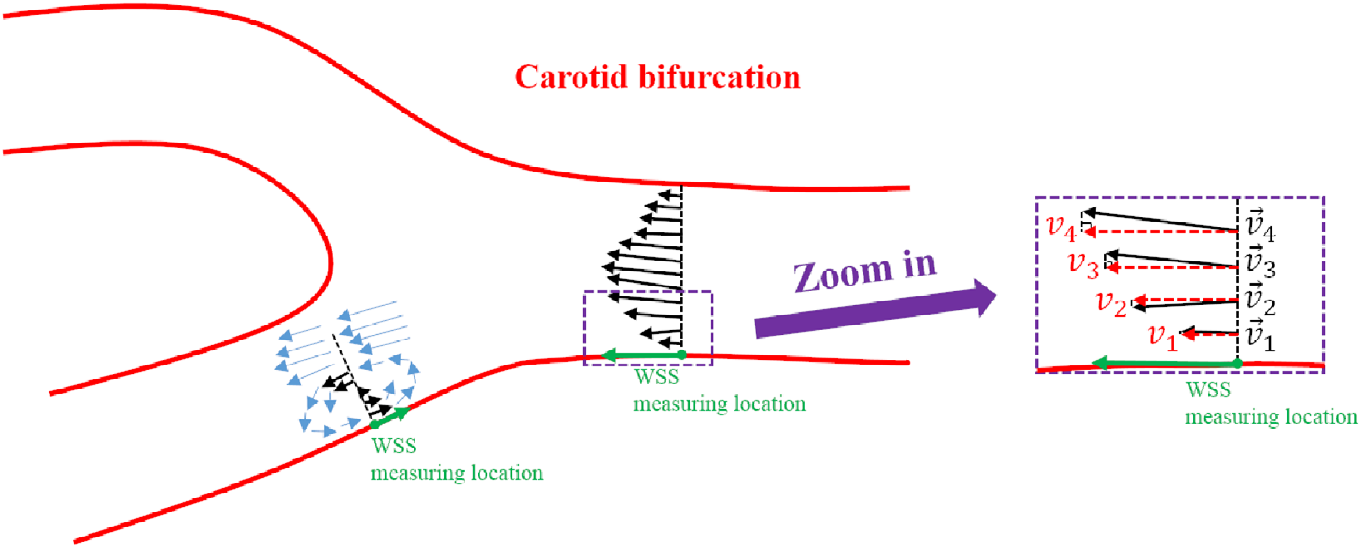
Wall shear stress measurements based on UVFI for nonlaminar flow; ${\vec{v}}_{1},{\vec{v}}_{2},{\vec{v}}_{3},\;\text{and}\;{\vec{v}}_{4}$, denoted by the black solid arrows, are the vector (true) velocities, which can be obtained by UVFI; *v*_1_, *v*_2_, *v*_3_, and *v*_4_, denoted by the red dashed arrows, can be derived from the vector velocities and are the velocities that should be used in the WSS estimations.

Based on UVFI, the WSS can be calculated no matter what kind of flow type and vessel shape they are. As shown in Figure 4, with the proposed UVFI technique, the vector WSS on the vessel wall as a function of time can be estimated by

(7) $$\vec{\tau }\left(t\right)=\mu \sum_{i=1}^{i=N}\frac{\vec{w}∙{\vec{v}}_{i}\left(t\right)}{{\Delta r}_{i}},$$

where *Δr*_*i*_ is the distance from the *i*th velocity measurement by UVFI to the WSS measurement location; ${\vec{v}}_{i}$ is the vector velocity shown as the black arrows in Figures 3 and 4; $\vec{w}$ denotes the direction of $\vec{\tau }$ and is a unit vector, which can be derived from the shape of the vessel wall shown in the grayscale image; and $\vec{w}∙{\vec{v}}_{i}\left(t\right)$ in Equation 6 denotes the derived velocities shown as the red arrows in Figure 4, which are the final used velocity arguments in the WSS calculation. Similarly, the WSS can also be estimated on the basis of the maximum value of velocity differences, which is formulated by

(8) $$\vec{\tau }\left(t\right)=\mu \times \max_{1\leq i\leq N}\frac{\vec{w}∙{\vec{v}}_{i}\left(t\right)}{{\Delta r}_{i}}.$$

Results of WSS estimation based on Equations 4, 5, 6, 7, 8 for different surrounding flows will be simulated in the next section.

## Simulation Results and Discussion

**Figure 5:**
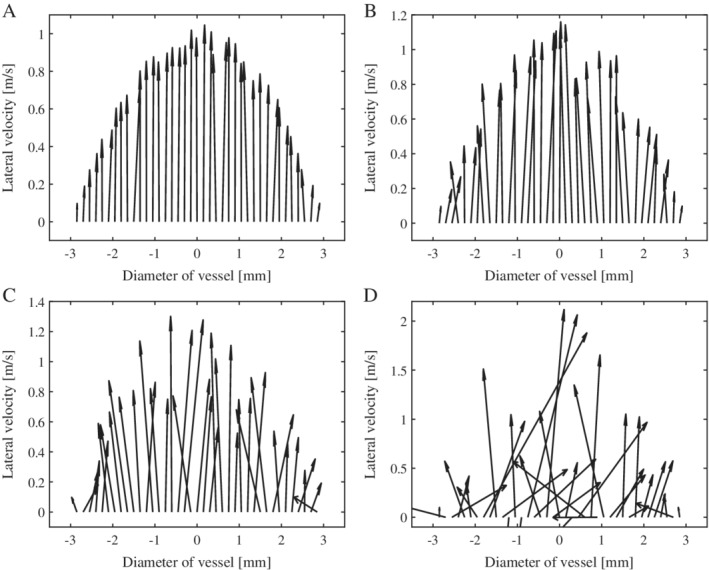
Vector velocity distribution profiles along the diameter of the vessel for turbulent flows with 4 different amplitudes: 5 cm/s (**A**), 15 cm/s (**B**), 30 cm/s (**C**), and 100 cm/s (**D**).

Nonlaminar flows exist widely in stenotic or tortuous vasculature, bi(tri)furcation, and even straight vessels with a high‐level Reynolds number (ie, Re >2300). The flow pattern also varies during different cardiac phases because of the pulsating wall and complicated hemodynamic changes. Clinical examples for specifics can be found in the next section. For simulating nonlaminar flows, a lateral velocity is considered the main velocity of blood flow, which has a parabolic distribution profile along the diameter of the vessel, and then velocity noise by a Gaussian distribution with zero expectation for both lateral and axial directions is added in the main velocity, as described in Table 1, where the nonlaminar flow is classified into different degrees of turbulence simulated by different amplitudes of the velocity noise. For each amplitude, 1000 realizations are simulated. Vector velocity distribution profiles are shown (Figure 5) as examples of realizations for 4 different amplitudes (5, 15, 30, and 100 cm/s).

**Table 1:** Parameters for the Different Degrees of Turbulence in the Simulation

Main Velocity (Lateral)	Amplitude of Velocity Noise (Gaussian)		Amplitude Increment	Vessel Diameter
	Lateral (Parabolic Peak)	Axial		
Parabolic profile with a peak value of 100 cm/s	5–100 cm/s for different degrees	5–100 cm/s for different degrees	5 cm/s	6 mm

**Figure 6:**
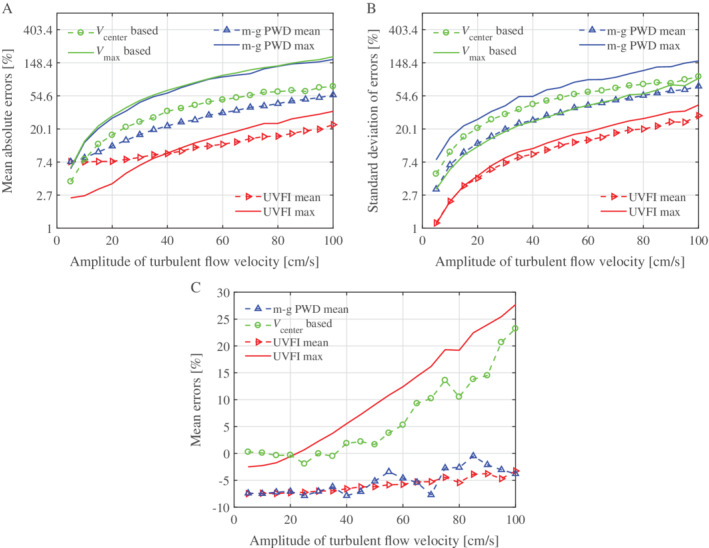
Errors and standard deviations of the simulated results based on UVFI and PWD, mean absolute errors (**A**), standard deviation of errors (**B**), and mean errors (**C**) for different amplitudes of turbulent flow velocities. Note that the values in **A** and **B** are shown in logarithmic (ln) coordinates. For *V*_center_ based, the velocity at the vessel center is used in Equation 4 to calculate WSS; *V*_max_ based, the maximum velocity along the vessel diameter is used in Equation 4 to calculate WSS; m‐g PWD mean, WSS is calculated by multigate PWD using Equation 5; m‐g PWD max, WSS is calculated by multigate PWD using Equation 6, multiplying by μ; UVFI mean, WSS is calculated by UVFI using Equation 7; and UVFI max, WSS is calculated by UVFI using Equation 8.

The WSS is calculated on the basis of the proposed UVFI and conventional US (PWD) using Equations 4, 5, 6, 7, 8. The diameter of the vessel is 6 mm, and the spatial interval is 0.15 mm in the WSS calculation. The mean (absolute) errors and standard deviations of 1000 simulations in percentages corresponding to each amplitude of velocity noise (different degrees of turbulence) for UVFI and PWD are plotted in Figure 6. The simulated true value of WSS is obtained from the lateral velocities by the following polynomial regression:

(9) $$f\left(x\right)=ax^{3}+bx^{2}+\mathrm{cx}+d,$$

where the coefficients *a*, *b*, *c*, and *d* are obtained by minimizing the mean square error between the predicted values and the lateral velocities. The WSR can be calculated by substituting the boundary condition (ie, vessel wall: *x* = 3 mm) to the derivative of Equation 9, ie, *f*′(*x*), and then multiplies by *μ* to get WSS, where the spatial interval is 0.015 mm (^1^/_10_ of the WSS measurement) in the regression to simulate the true value. For each realization with the corresponding degree of turbulence, there is a simulated true value, which will be used as the reference for calculating the errors (Figure 6) for different WSS‐measuring techniques.

It can be seen that the overall performance of UVFI is better than that of multigate PWD, which is also better than that of maximum velocity PWD‐based WSS measurement using Equation 4. The calculating errors are more stable (low standard deviations and low errors for a high degree of turbulence) for mean value–based measurements and are relatively low for the low degree of turbulence (particularly for UVFI) for maximum value–based measurements. The errors are significantly reduced by the mean value–based UVFI (UVFI mean in Figure 6A), particularly for the high degree of turbulence compared to other techniques. However, it seems that there is a fixed underestimating error for both the multigate PWD and UVFI mean value–based measurements (m‐g PWD mean and UVFI mean in Figure 6C). This error is probably caused by the inadequate spatial sampling rate, as the velocities in Equations 5 and 7 could be far from the WSS measurement location. This could give rise to a lower WSS measurement result because the velocity gradient becomes smaller and smaller when the location moves from vessel wall to inner, as the parabolic profile is assumed.

## Clinical Examples and Discussion

This preclinical study aimed to evaluate the correspondence between the flow behaviors as shown by UVFI with the expected WSS values in the CB as known from previous studies.^14^, ^15^ Specifically, the relationship between CB geometry and flow patterns was evaluated in 30 vessels of healthy participants (15 men and 15 women; mean age, 39.5 years; age range, 23–59 years) previously enrolled for another study.^45^ In comparison to the previous study, from the UVFI raw data of the healthy volunteers stored into the system, new information was extracted, without the need to resubmit them to a new examination. Five patients (4 men and 1 woman; mean age, 70.4 years; age range, 65–75 years) affected by different degrees of internal carotid stenosis were enrolled to evaluate the ability of UVFI in measuring the expected higher WSS values at the stenosis level. Further clinical research would be focused on other pathologic conditions. The study was approved by the Ethical Committee of the Fondazione Istituto di Ricovero e Cura a Carattere Scientifico, Policlinico San Matteo, and written informed consent was obtained from all participants.

The US examinations were performed with the Resona 7 system equipped with L9‐3U and L11‐3U linear array transducers. The flow within a selected region of interest is analyzed by the system with UVFI (V Flow option of Resona 7), a non–real‐time technique.^32^ An interleaved sequence of plane waves at different angles and focused waves is sent into the body for 1.5 seconds, thereby performing the insonification of the flow in a single shot and allowing the examination of at least a single cardiac cycle. The frame rate is dependent on the pulse repetition frequency for an updated system and was around 500 to 600 Hz for different cases of our acquisitions in this study. The acquired data are immediately automatically reprocessed by the system, generating a sequence of hundreds of images displayed dynamically in slow motion in half a minute around a video clip. The high temporal resolution with a dynamic display offers detailed visualization of the flow through the cardiac cycle, allowing distinction of the different flow components and their extensions and durations.^45^ The UVFI technique enables a quantitative analysis of various hemodynamic parameters on the stored raw data. In particular, the updated system supports a series of 6 WSS measurements selected by the operator along the vessel walls from the playback video clip. The measurement was made by overlapping the reference midline of a dedicated caliper over the intimal layer of the vessel wall and by adjusting a correction line perpendicular to the vessel wall to obtain an accurate evaluation of the shear rate. Maximum and mean WSS values between a time interval (cardiac cycle) for each selected point are available. All of the acquisitions and measurements were obtained by A.G., a radiologist with more than 25 years of experience in the Doppler field. Scan sequences of the CB were obtained in the supine position after resting of almost 10 minutes. The entire process, from acquisition to WSS measurements, takes about 3 minutes for each carotid artery (Table 2).

**Table 2:** Ultrasound Vector Flow Imaging Flow Chart and Time Consumed for WSS Measurements

Acquisition Phase	Automatic Data Reprocessing	Video Clip Playback	Velocity Measurements	WSS Measurements^a^
1.5 s	≈30 s	≈30–60 s	≈30 s	≈60 s

a Two series of 6 measurements.

The reference values for WSS, already established in the literature, were used to evaluate the presence of normal or abnormal WSS values: the normal maximum WSS in arteries was considered to be between 1 and 7 Pa^15^; a local mean WSS lower than 0.4 Pa was considered abnormal and to promote plaque development.^14^ The detection of at least a single abnormal WSS value along the vessel wall was considered a positive result for the test. A second measurement was taken in areas of abnormal values to confirm the finding. In this preclinical study, intraoperator and interoperator variability were not evaluated because of the small sample of examined participants.

Ultrasound vector flow imaging showed the presence of nonlaminar flow during the systolic deceleration phase in the most volunteers (28 of the 30 cases). In 8 of them, UVFI detected a counter‐rotating helical trajectory. Both participants with laminar and helical flows (33.3%) did not have abnormal WSS values. In the remaining cases, the presence of reversed (15 of 30 [50%]) and complex (5 of 30 [16.6%]) flows in the internal carotid artery were found. A strong correlation (*r* = 0.83) between the internal carotid artery enlargement and the presence of complex flow was found: the more relevant the diameter, the more disturbed the flow. The flow patterns appeared to be much more sensitive also to anatomic changes in the branch flow division (Table 3). Large areas of reversed and complex flows resulted in lower WSS values and extensive areas of abnormal WSS. The study did not consider the accuracy in detecting and measuring the WSS values with UVFI because it should be analyzed by comparing the WSS finding with computational fluid dynamics.

**Table 3:** Ultrasound Vector Flow Imaging Analysis of Flow Patterns in 30 CBs of Healthy Participants

Laminar Flow^a^	Rotational Flow^b^	Reversed Flow^c^	Complex Flow^d^
2	8	15	5

a Streamlines move forward along the vessel axis.

b Streamlines rotate around an axis of flow in the forward direction.

c Streamlines move back to reversed flow into a separation zone.

d Streamlines show multidirectional vectors or curl back on themselves by a swirling motion.

Evidence suggests that flow dynamics, in addition to systemic risk factors, contribute to the development of atherosclerosis. Consequently, an accurate assessment of hemodynamic changes promoting carotid plaque development is critical in cardiovascular risk prevention. The potential value of WSS in evaluating hemodynamic changes in the carotid artery as a biomarker for cardiovascular risk stratification has been recently proposed.^46^ A physiologic intermediate shear stress protects against atherogenesis via a trimolecular complex expressed on endothelial cells and other receptors, which converts mechanical stress into a biochemical response by suppressing prothrombotic tissue factor activity as well as anti‐inflammatory activation of endothelial cells.^47^, ^48^, ^49^ By contrast, low and oscillatory shear stress is able to reduce nitric oxide production and to induce proinflammatory mediator synthesis.^48^ High WSS is considered a determinant of increased lipid (necrotic core) intraplaque accumulation, which acts in combination with plaque structural stress of the overlying fibrous cap to promote plaque failure.^50^, ^51^ However, the prerequisites for a precise WSS measurement are the ability to analyze the blood flow patterns, to detect transient flow disturbances, and to identify the recirculation regions where the WSS variations are calculated.

The high–frame rate UVFI technique (V Flow option) has been shown to answer the above requirements in several articles.^30^, ^45^, ^52^, ^53^, ^54^ The most relevant advantage is the ability of UVFI to outline a detailed depiction and quantification of complex flows, given that local hemodynamic variations strongly influence the WSS.^55^, ^56^

**Figure 7:**
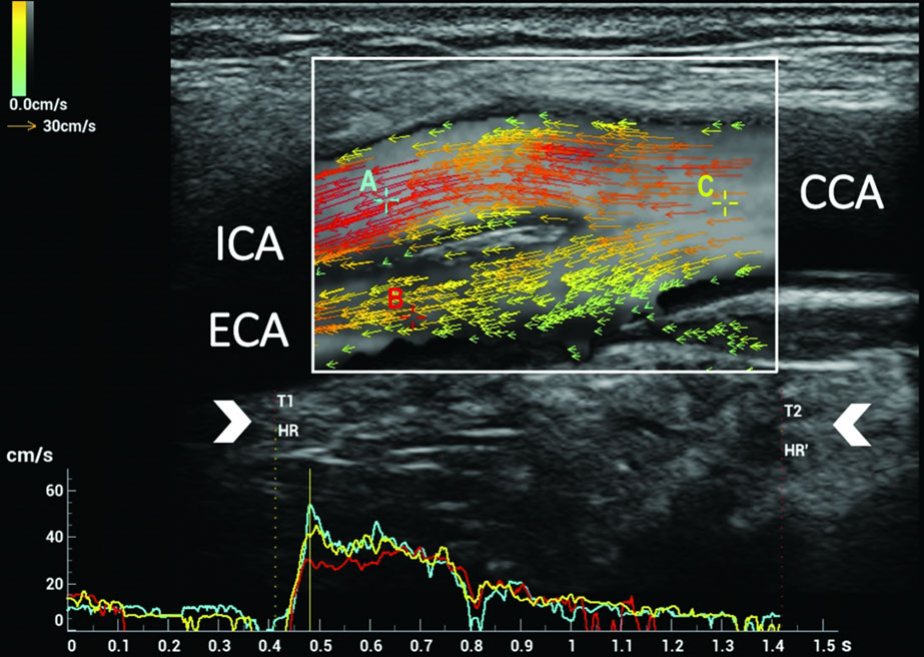
Ultrasound vector flow imaging of laminar flow in the CB. In the upper part, a single image, corresponding to the systolic peak, was extracted from the 900 images of the cine loop acquired in 1.5 seconds. The system measures the speed and direction of all blood cells flowing through every point of the selected region of interest. The flow is represented by several color arrows. The color and size of the arrows allow visual quantification of flow velocity in the 2D space. The arrows are randomly distributed and flow according to their velocities. At a static image (a single frame), different concentrations of the arrows in the lumen could be seen. In the lower part, the flow velocity‐versus‐time curves were measured in 3 locations of the CB: internal carotid artery (ICA) curve (A) displayed in light blue, external carotid artery (ECA) curve (B) in red, and common carotid artery (CCA) curve (C) in yellow: the time interval (T1–T2) corresponding to the cardiac cycle duration (arrowheads) can be selected manually on the acquired row data video clip, thus allowing a correct subsequent WSS measurement. In the updated version, the HR and HR′ (as shown in the image) allow the selection of the corresponding cardiac cycle duration automatically or manually.

A typical example of UVFI in the CB is shown in Figure 7. The flow is represented by several color arrows showing the different velocities, magnitudes, and directions at every point of the vessel. The color and length of the arrows allow visual quantification of the flow behavior. In particular, green arrows denote low velocities; yellow and orange denote medium velocities; and red denotes high velocities; the longer the arrows, the faster the flow.^52^ The flow characteristics can be evaluated visually to assess the flow pattern by considering the vectors’ directions and lengths.

Using UVFI, the WSS measurements at different locations can easily be estimated, as vector velocities are already known, and the calculation is done by finding the corresponding velocity components with the reference of the vascular shape. The correct positioning of the caliper for the point‐by‐point WSS measurement is allowed by B‐mode identification of the vessel wall and the simultaneous color Doppler (intensity mode) display of the flow in the background, which outlines the endothelial profile or the plaque surface. In a recent study, UVFI resulted in a simple, rapid, and feasible imaging method, with excellent interobserver reliability in assessing WSS values of the common carotid artery in healthy adults.^57^ The different WSS findings related to laminar, rotational, reversed, complex, and turbulent flows are condensed in the following clinical cases.

**Figure 8:**
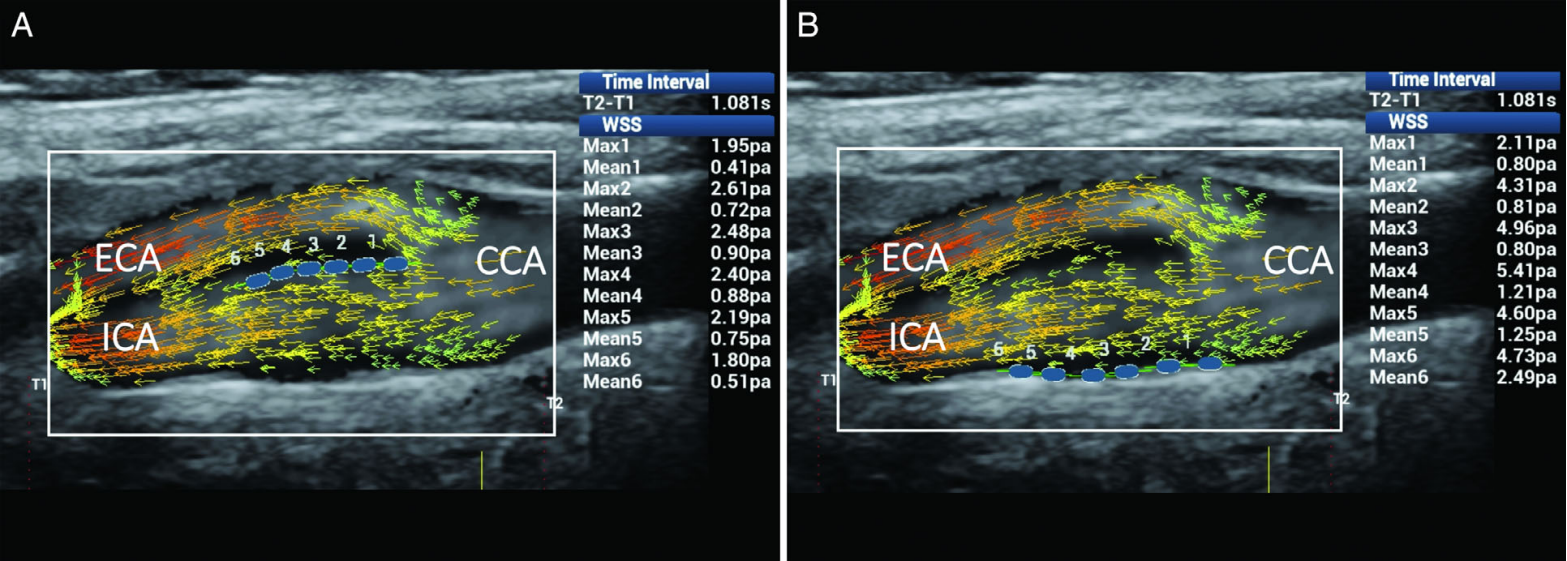
Mainly laminar flow in the CB. Ultrasound vector flow imaging shows the vector direction remaining parallel to the vessel walls. Wall shear stress measurements (blue dots) gave maximum and mean values within the normal range: in particular, along the flow divider, 1.80 to 2.61 and 0.41 to 0.90 Pa, respectively (**A**), and along the opposite wall from the flow divider, 2.11 to 5.41 and 0.80 to 2.49 Pa (**B**). CCA indicates common carotid artery; ECA, external carotid artery; and ICA, internal carotid artery.

The first of them is shown in Figure 8. It concerns mainly laminar flow in which the direction of velocity vectors remains parallel to the vessel walls, and the shear stresses maintain their magnitude within a range of normal values.

**Figure 9:**
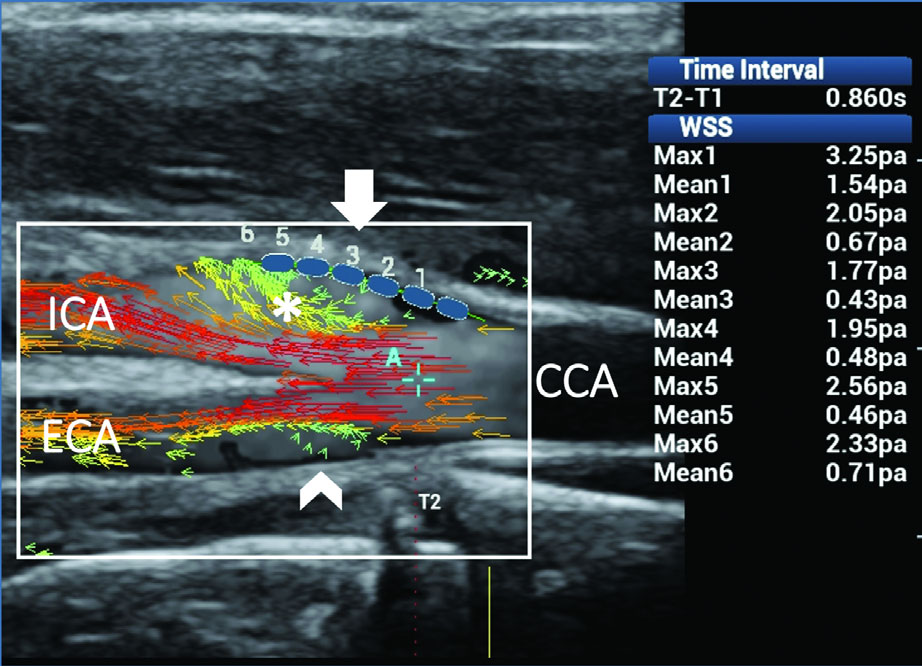
Rotational flow in the internal carotid artery (ICA). Ultrasound vector flow imaging shows flow layer separation in both the ICA sinus and external carotid artery (ECA). High‐velocity red vectors move along the 2 sides of the flow divider. Low‐velocity short green vectors rotate around an axis of flow along the outer wall while moving forward in the ICA (asterisk) and move back in a small recirculation area along the outer wall in the ECA (arrowhead). Wall shear stress measurements (blue dots) along the outer wall of the ICA (arrow) gave maximum and mean values within the normal range (1.77–3.25 and 0.43–1.54 Pa, respectively), thus confirming the steady shear stress related to the helical flow. CCA indicates common carotid artery.

**Figure 10:**
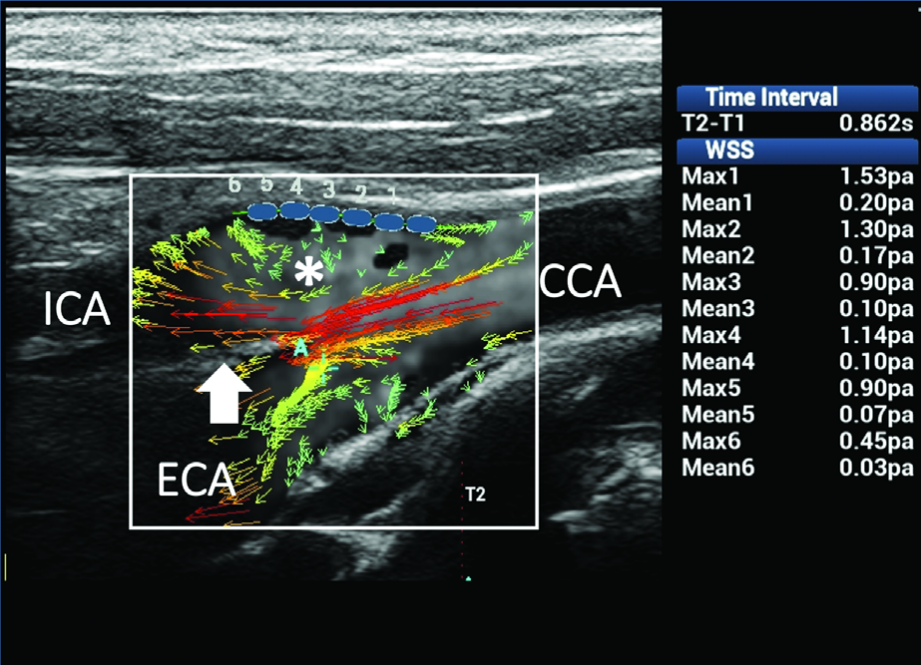
Reversed flow in the CB. Ultrasound vector flow imaging shows high‐velocity red vectors moving forward along the flow divider in the internal carotid artery (ICA; arrow). Some low‐velocity green vectors move back on the opposite wall to the flow divider in the ICA sinus (asterisk). Wall shear stress measurements (blue dots) at that level gave about normal maximum values (0.90–1.53 Pa) in 5 of the 6 samples and abnormal mean values (0.03–0.20 Pa) in all of the sample points as a consequence of the unsteady shear stress. CCA indicates common carotid artery; and ECA, external carotid artery.

The second and third clinical cases are related to disturbed flows caused by changes of the lumen diameter and resulting in flow layer separation with rotational or reversed flows. When vectors rotate around an axis of flow, their directions move forward into the flow layer separation area, thus resulting in steady shear stress and consequently normal WSS values (Figure 9). By contrast, in the presence of reversed flow, abnormal mean WSS values, due to the unsteady shear stress, can be found on the opposite wall from the flow divider, where secondary flows usually develop (Figure 10).

**Figure 11:**
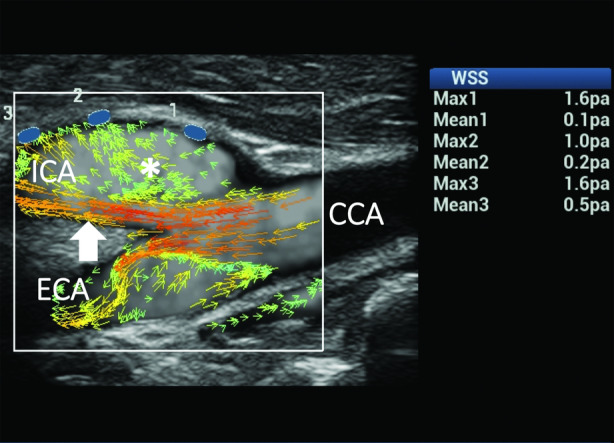
Complex flow in the CB. Ultrasound vector flow imaging shows changes of direction and velocity of the flow layers due to vessel dilatation, resulting in the development of complex flow in both the internal carotid artery (ICA) and external carotid artery (ECA). High‐velocity red vectors flowing near the flow divider in the ICA (arrow) and a wide area of multidirectional low‐velocity green vectors (asterisk) are shown in the ICA sinus. Wall shear stress measurements on the outer wall (blue dots) of the ICA gave normal maximum values (1.0–1.6 Pa) in all of the samples and abnormal mean values (0.1–0.2 Pa) in 2 of the 3 sample points. CCA indicates common carotid artery.

**Figure 12:**
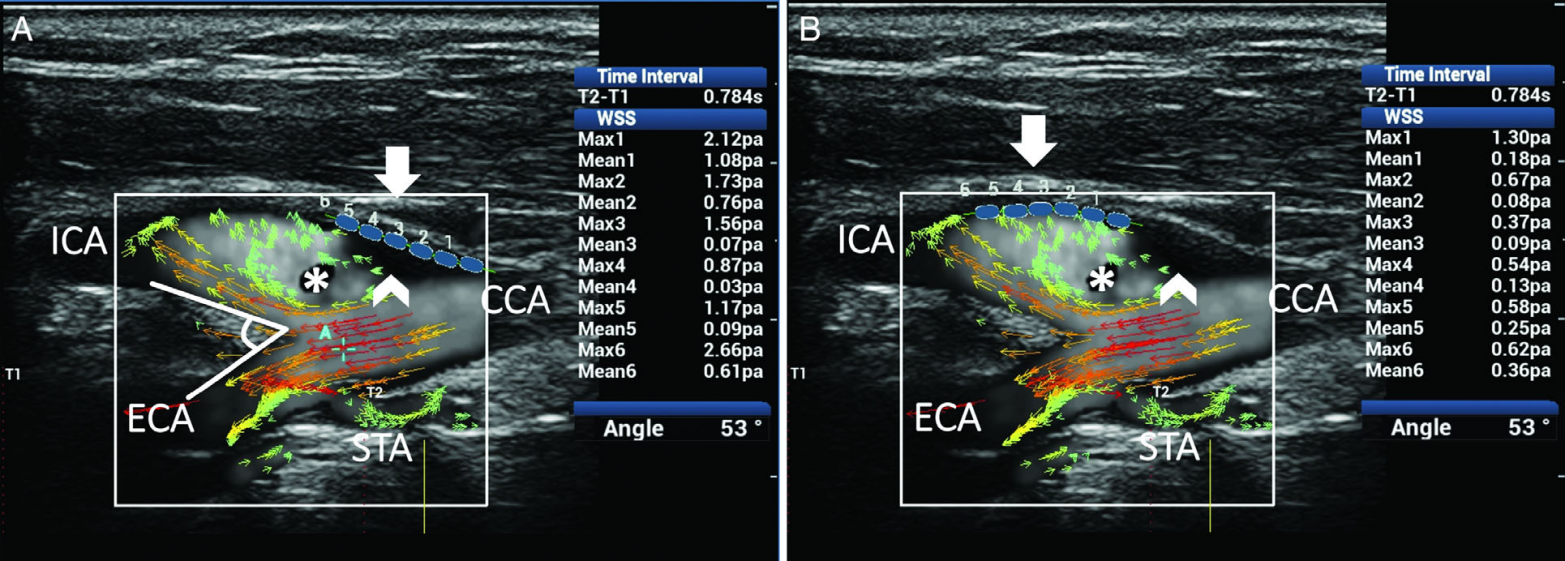
Complex flow in a CB with a 53° bifurcation angle. Ultrasound vector flow imaging shows the development of complex flow due to vessel geometry and bulb–internal carotid artery (ICA) sinus dilatation. High‐velocity red vectors flowing near the divider in the ICA and a wide area of recirculation with low‐velocity green vectors moving in a counter eddy (asterisk) are shown in the outer side of the vessel. Areas in the lumen with no vector arrows and with no background speckle (asterisk and arrowhead) correspond to a flow so slow (near 0 velocity) that it cannot be detected by the system in this specific image or are not displayed because of the setting value of gain selected by the operator. Two series of 6 WSS measurements (**A** and **B**) on the outer wall (blue dots) of the bulb and ICA both gave abnormal maximum values (<1.0 Pa; range, 0.37–0.87 Pa) in 6 of 12 sample points and mean values (<0.4 Pa; range, 0.03–0.36 Pa) in 9 of 12 sample points. CCA indicates common carotid artery; ECA, external carotid artery; and STA, superior thyroid artery.

The last 2 cases consider the sudden changes of direction and velocity of the flow layers caused by the vessel geometry variation of the CB, which favors the development of complex flows (Figures 11 and 12). Ultrasound vector flow imaging displayed the layers’ detachment from the opposite wall from the flow divider, with vortices and counter eddies in the carotid sinus. Abnormal mean and maximum WSSs were found at the level of flow instability and slow fluid movement. These described findings are in accordance with many existing studies^14^, ^15^, ^58^, ^59^, ^60^ reporting that unstable flows and low shear stresses are mainly around the outer walls of the bifurcations, where lesions also occur most frequently.

In our study, atherosclerosis was found to generate different changes in WSS depending on the different degree of stenosis. A few clinical cases may explain the scenario.

**Figure 13:**
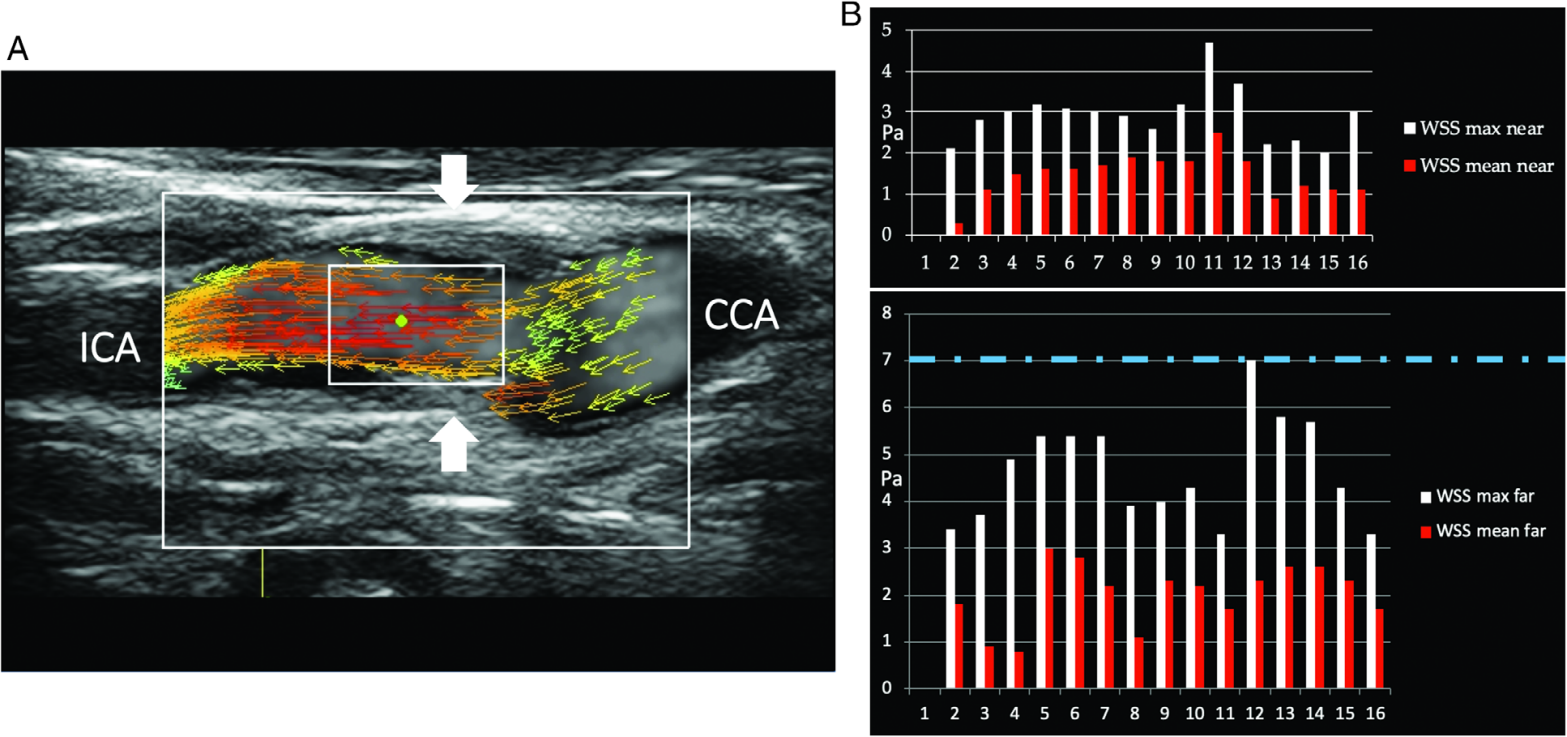
Mild carotid stenosis. **A**, Ultrasound vector flow imaging shows high‐velocity red vectors moving parallel to the vessel walls inside the stenosis (arrows). **B**, Wall shear stress point‐by‐point measurements on both the near and far walls had maximum and mean values (2.0–7.0 and 0.8–3.0 Pa, respectively) mainly within the normal range (dashed line corresponds to the maximum normal value). CCA indicates common carotid artery; ECA, external carotid artery; and ICA, internal carotid artery.

**Figure 14:**
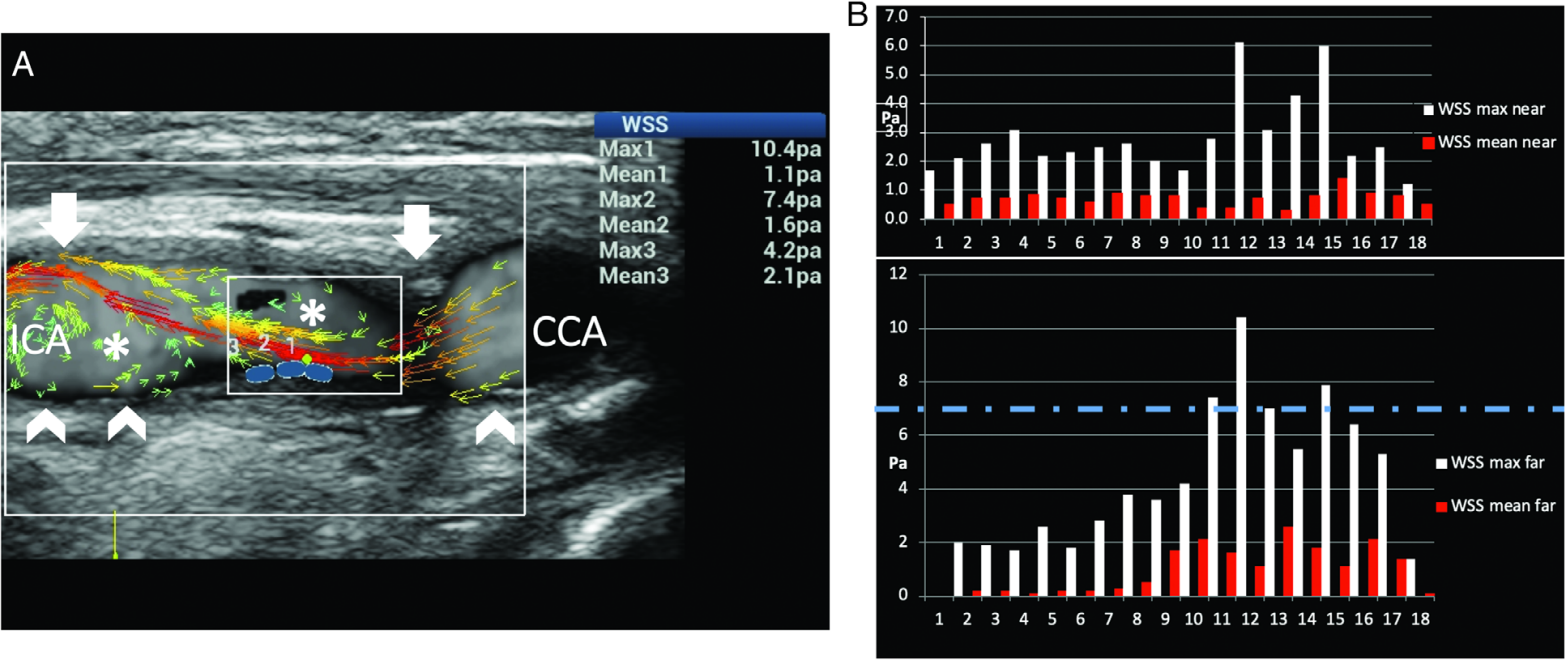
Moderate carotid stenosis. **A**, Ultrasound vector flow imaging shows high‐velocity red vectors at the level of and distal to the stenosis (arrows), associated with turbulent flow in the poststenotic area (asterisk). **B**, The graphs obtained by measuring WSS point by point along the near and far vessel walls show a high WSS maximum value of 10.4 Pa (dashed line corresponds to the maximum normal value) in the poststenotic area of the far wall (blue dots on **A**). Abnormal values low mean WSS values (0.1–0.3 Pa) were detected at the proximal edge of the plaque (0.1 Pa) and distally (0.1–0.3 Pa) on the far wall (arrowheads on Figure **A**). CCA indicates common carotid artery; and ICA, internal carotid artery.

**Figure 15:**
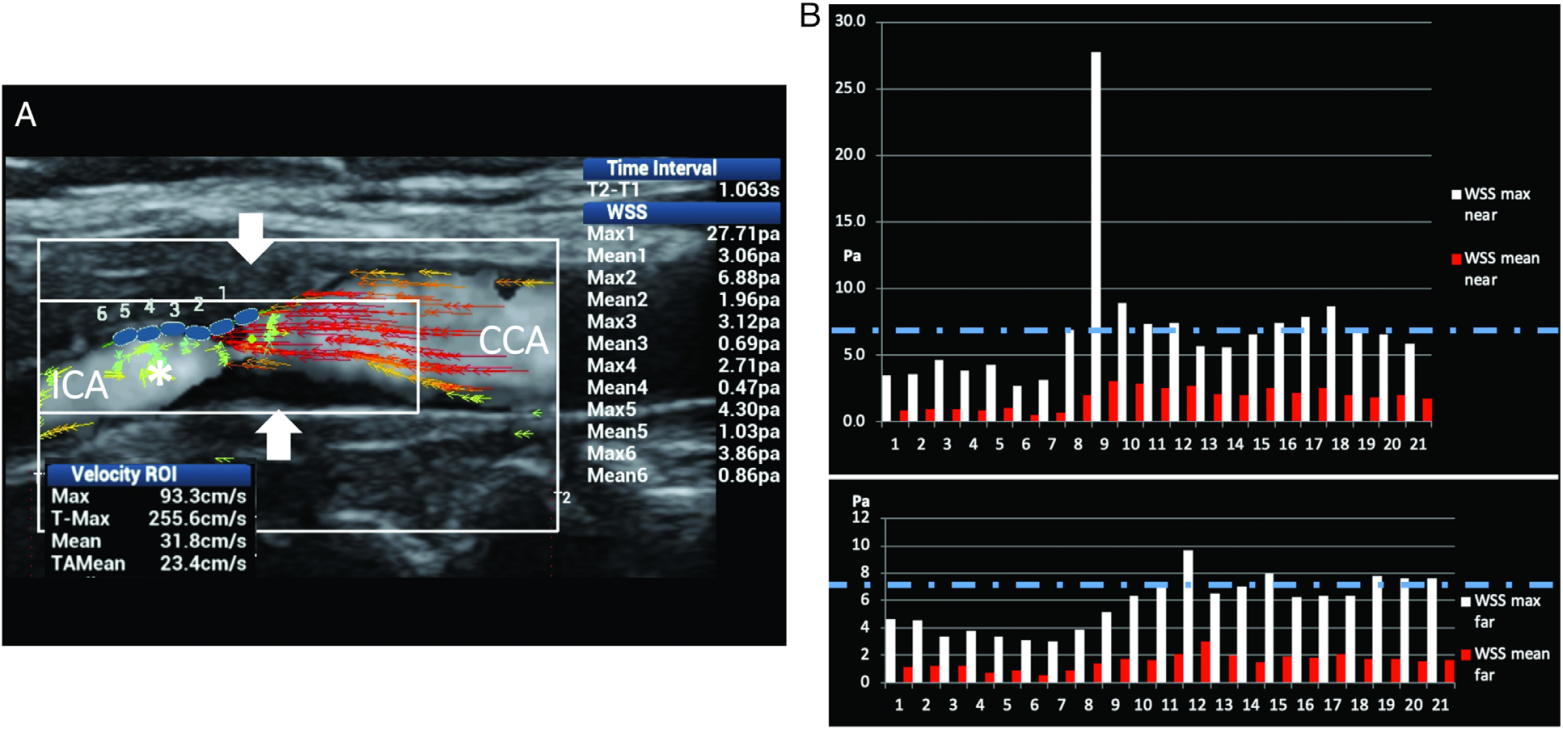
Substantial carotid stenosis. **A**, Ultrasound vector flow imaging shows very high‐velocity red vectors (maximum velocity along time in the region of interest, 255 cm/s) at the level of the stenosis (arrows) and substantial turbulence distally to the stenosis (asterisk). **B**, The graphs obtained by measuring WSS point by point along the near and far vessel walls show very high WSS maximum values of 27.7 and 9.69 Pa, respectively (dashed line corresponds to the maximum normal value) at the level of the stenosis. CCA indicates common carotid artery; and ICA, internal carotid artery.

In mild‐degree vessel stenosis (Figure 13), WSS measurements on both the near and far walls had maximum and mean values within the normal range. On the other hand, higher shear stress values have been observed in regions where moderate and substantial vessel stenosis promotes turbulent flow or an increased flow velocity (Figures 14 and 15). Measuring the WSS point by point along the anterior and posterior vessel walls showed the shear stress variations on the plaque surface and at the 2 edges of the plaques. In particular, very high maximum values at the level of the stenosis and abnormal low mean shear stress values have been detected at the edges of the plaques. Our findings confirmed what was expected from the literature: the presence of high and low shear stress values is frequently accompanied by unstable flow conditions, such as complex and turbulent flows, regions of blood recirculation, and areas with a low momentum of fluid.^15^

There were some limitations to this study. First, the simulations did not cover every possible scenario of the flow behavior. Second, the examinations were conducted by a single operator, and intraoperator and interoperator variability was not evaluated. Lastly, the small number of cases did not allow a correct analysis of the performance of the WSS measurements based on UVFI. However, this was a preclinical study aimed at assessing the correspondence between the presence of nonlaminar flows and abnormal WSS values in the CB.

## Conclusions

Local hemodynamic alterations and vessel diameter variations have been claimed to be the factors leading to an abnormal endothelial response through WSS alterations. The evaluation of WSS in clinical practice may be useful in the investigation of numerous vascular pathophysiologic conditions such as CB atherosclerosis, neointimal hyperplasia formation in arteriovenous fistulas and endovascular stents, and aneurysm formation. The abilities to accurately analyze blood flow characteristics, to detect transient flow disturbances, and to depict the oscillating flow in the recirculation areas would be essential prerequisites for a diagnosis based on a WSS assessment in atherosclerotic disease.

Ultrasound vector flow imaging, which provides directional information on velocities and more excellent temporal and spatial resolution, seems to be able to estimate WSS accurately. The simulated results showed that the UVFI‐based WSS measurements had much fewer errors than the conventional (multigate) PWD‐based results, particularly for high‐degree turbulent flows. Further studies are necessary, but if the preliminary findings are confirmed, UVFI could be added to current US vascular examination protocols for the patient's risk stratification, thus allowing clinical management in the early stage of atheromasia.

## Disclosure

Dr Goddi is consultant for Esaote, Shenzhen Mindray Bio‐Medical Electronics Co., Ltd., and has been consultant for SuperSonic Imagine. Dr Calliada is consultant for Hitachi Medical Systems Europe, Shenzhen Mindray Bio‐Medical Electronics Co., Ltd., and Toshiba Medical Systems Europe. Dr Bortolotto is consultant for Bracco Imaging Italia, and Doc. Congress. Dr Du, Ms Shen, Mr Dell'Era, and Dr Zhu are employed by Mindray.
